# Enhanced Broadband RF Differential Rectifier Integrated with Archimedean Spiral Antenna for Wireless Energy Harvesting Applications

**DOI:** 10.3390/s19030655

**Published:** 2019-02-05

**Authors:** Mohamed Mansour, Xavier Le Polozec, Haruichi Kanaya

**Affiliations:** 1 Graduate School of Information Science and Electrical Engineering, Kyushu University, Fukuoka 819-0395, Japan; kanaya@ed.kyushu-u.ac.jp; 2 Electronics Research Institute, Microelectronics Department, Giza 12622, Egypt; 3 Engagement Practice IP Transport, Ericsson, 91348 Massy, France; xavier.le-polozec@ericsson.com

**Keywords:** Archimdean spiral, balun, differential rectifier, rectenna, wireless power transfer

## Abstract

This work addresses the design and implementation of a broadband differential rectifier (DR) combined with an Archimedean spiral dipole antenna (ASDA) for wireless power harvesting at low incident power densities below 200 μW/cm${}^{2}$. The proposed design exhibits an improved RF-DC conversion efficiency over a wide frequency range from 1.2 to 5 GHz. This frequency band is associated with several wireless communication services, for instance, ISM, WLAN, 5G, LTE, and GPS applications. The receiving planar ASDA exhibits circular polarization and has an average measured gain of 4.5 dBi from 1.2 to 5 GHz. To enable a wide operating bandwidth, the rectifier circuit is constituted by two architectures, designated A and B. Each scheme is designed to harvest power efficiently across a specific bandwidth. The optimal performance of both rectifiers are obtained using the nonlinear harmonic-balance simulations. The antenna–rectifier integration yields a compact rectenna with a high-efficiency performance over the intended bandwidth from 1.2 to 5 GHz for an input power of 9 dBm and terminal load resistance of 1 kΩ. The total measured RF-DC conversion efficiency is maintained above 30% across the entire frequency range with a peak value of 61% achieved at 1.2 GHz. In comparison with similar architectures, the proposed rectenna maintains a stable output efficiency despite the wide fluctuations in the input frequency and also has a minimum footprint size (58 × 55 mm${}^{2}$).

## 1. Introduction

In the past few decades, owing to the explosive growth of the microelectronics industry and semiconductor technology, electronic products, which have not only become increasingly miniaturized but also increasingly complex, have played a very important role in transforming the human society. The emergence of numerous electronic devices, such as laptops and mobile phones, has changed human lifestyle. Energy consumption is a significant challenge for the electronic designer community. The growing demand for a powerful electronic system with declining costs has led to faster, smaller, and more reliable integrated circuits that require less power. Owing to the tremendous achievements in low-power radio transceivers, many low-power wireless sensors that consume only several microwatts have been developed. More recently, researchers have developed a method for designing picowatt radio chips [1]. The power requirement declination has encouraged researchers to implement an independent wireless node that could obtain energy from the surrounding environment via several energy conversion techniques, for instance, solar, wind, vibration, thermal, and RF energy harvesting (RF-EH) [2]. Energy harvesting from ambient energy sources can potentially reduce the dependence on the supply of grid or battery energy, providing many attractive benefits for the environment and deployment [3].

RF-EH has become a well-known method for converting the received electromagnetic energy into DC power. RF-EH provides many promising advantages over other energy conversion methods owing to its predictable and stable nature, low cost, and small form factor [4]. The RF-EH technology has the potential to be an alternative power source for various applications such as wireless sensor networks (WSNs) [5], wireless body networks [6], and wireless charging systems [7]. The harvesting of ambient energy to power WSNs has been extensively investigated over the last decade for prolonging the lifetime of wireless devices [8,9,10,11,12,13,14,15,16]. A comprehensive survey on the recent RF-EH technologies has been done in the work of [17,18] and a brief explanation about the energy requirement of low-power wake-up receivers has been provided with an introduction to EH in [19]. The research goals of this field are to enable self-powered electronic nodes and power energy-constrained wireless networks.

Most of the wireless communication systems ranging from 3G, 4G, and LTE mobile communication networks emit power into the environment, and similar to a perpetual electromagnetic energy source. Several frequency bands are assigned to each communication network; therefore, the desired electromagnetic spectrum is shared and allocated across multiband and even broadband frequencies. In addition, scavenging power from the environment is time-varying and limited in most circumstances, which stipulates a new design constraint on the energy usage along over time. Consequently, it is preferable to implement a wideband energy harvesting circuit, which could operate efficiently and collect the power from different available frequency bands.

In the literature, several research studies have reported the demonstration of wide bandwidth rectifiers as well as the differential topology design. In the work in [20], a distributed array of antennas with an optimized power-management circuit was introduced to increase the harvested power and efficiency. It offered a significant advantage in terms of the wide bandwidth, efficiency, and value of received power density. The system employed an array of circular polarized antennas, with each one connected to a rectifier circuit. Consequently, it had the disadvantages of a large footprint and high-volume cost. A design of UWB rectifiers that use non-uniform transmission lines for broadband matching was presented in [21]. An RF-DC conversion efficiency of more than 60% was obtained for an octave frequency band from 470 to 860 MHz at 10-dBm-input power for a four-diode rectifier configuration. A two-diode charge pump printed on a flexible substrate has achieved an efficiency of more than 33% for an ultrawide frequency band between 250 MHz and 3 GHz. A frequency-selective surface (FSS) absorber introduced in [22] was used for ambient RF-EH. The FSS unit cell was constructed with built-in channeling features to combine the collected power from multiple unit cells. The FSS absorber was then integrated with a matched full-wave rectifier. The complete rectenna prototype showed a 61% RF−DC power conversion efficiency.

In the study reported in [23], a differential microstrip antenna with a gain of 8.5 dBi and bandwidth of 135 MHz was designed and used for RF-EH applications. A peak efficiency of 65.3% was achieved at a received power of 2.19 dBm at 980 MHz. A differential rectifier (DR) with high RF−dc conversion efficiency over an extended input power range was also investigated by using a resistance compression network (RCN) [24]. It could reduce the variation range of the rectifier input impedance, which changed with the input power levels, and a better impedance matching could be realized. The measured results showed that the RF-DC conversion efficiency was maintained above 50% and 70% in the input power range of 5.5–33.1 and 13.5–31.3 dBm, respectively.

Most of the previously proposed circuits were hindered by the multilayer fabrication, large circuit size, and limited bandwidth of the efficient rectifier designs.

There are two challenges in the realization of a wideband energy harvesting circuit. First, the receiving antenna should exhibit a wideband characteristic and show a good radiation performance in the desired band. A spiral antenna is the most suitable antenna type that can satisfy these requirements [25,26,27,28,29]. Second, the rectifier circuit should exhibit an efficient wideband performance. In this study, the DR topology discussed in [30] is selected. However, that topology used a balance–unbalanced transmission line (BALUN) for achieving appropriate interfacing with the unbalanced RF source. In this work, BALUN is avoided. Moreover, it is combined with an additional rectifier architecture to enhance the bandwidth. The DR topology offers a balanced DC output voltage, which can directly supply the load without any conditioning or power management circuit. Hence, it avoids the use of any additional components or complex circuits. One more advantage is the simple interfacing between the proposed spiral antenna and DR without port transformations.

Summarizing, the main contributions of this study are:

- A wideband circular-polarized planar antenna connected with a feeding BALUN was designed and fabricated, and the antenna radiation characteristics were obtained.
- Two rectifier architectures were constructed separately and then combined to improve the overall rectifier response.
- The aforementioned rectifiers were optimized simultaneously with the ASDA to realize the complete rectenna system.

A rectenna prototype was fabricated, and the experimental performance of the rectifier circuit and antenna was characterized and compared with the electromagnetic simulation results. The remainder of the manuscript is organized as follows: the ASDA and BALUN design are discussed in Section 2. The rectifier design is described in Section 3. Section 4 is devoted to the experimental performance and measurement set-up. The conclusions are drawn in Section 5.

## 2. ASDA and BALUN Design

Many wireless communication systems require antennas to operate across a broad bandwidth. As energy harvesting tends to collect much spectrum power, it is preferable to design a wideband antenna. A broadband antenna usually requires a structure that does not have abrupt changes in the geometry but instead have a smooth geometry [31]. In this section, we are going to focus on the design of the ASDA and its feeding topology. The feeding architecture is implemented using a wideband-tapered microstrip BALUN microwave component.

### 2.1. BALUN Design

A wideband transition from the coaxial line to the parallel strip line is designed to feed the antenna. In practical applications, low-profile spiral antennas suffer from the lack of suitable feeding methods. A vertical BALUN is the most used technique for improving the matching of the antenna input impedance to an excitation source impedance of 50 Ω. The BALUN was designed by using electro-magnetic (EM) simulator (ADS, Keysight, Santa Rosa, CA, USA).

**Figure 1:**
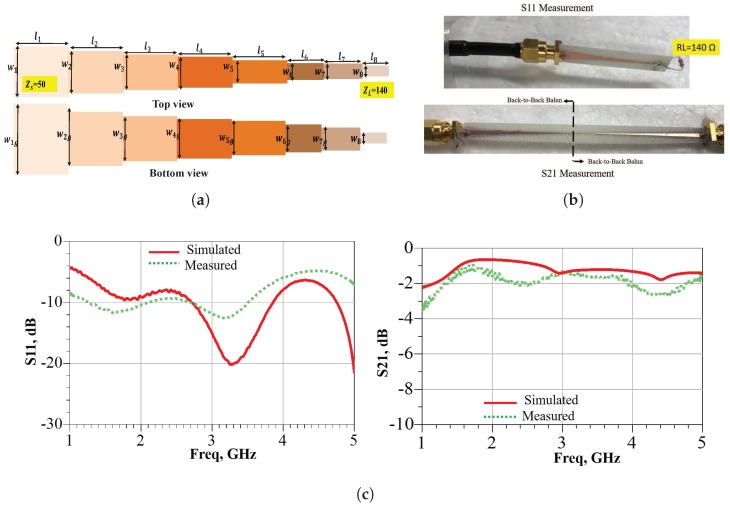
Fabricated balun layout and the measurement set-up for the performance characterization. (**a**) Photograph of the top and bottom view of the Balun layout; (**b**) Measurement set-up for $S_{11}$ and $S_{21}$; (**c**) Comparison between the measured and simulated data.

An integrated BALUN is a microstrip-tapered transmission line [32], as shown in Figure 1a. The impedance transformation is performed by a step decay in the transmission line width from the source (50 Ω) to the load (140 Ω). The 140-Ω-impedance represents the input real resistance of the ASDA.

The experimental measurement set-up is demonstrated in Figure 1b. For $S_{11}$ evaluation, a single BALUN is terminated with 140 Ω and its port is connected to a coaxial cable (50 Ω). Then, a single-port measurement is computed. The insertion loss measurement is slightly different owing to the requirement of a two-port calibration. Consequently, another BALUN is connected back-to-back to constitute a balanced two-port network. The resultant BALUN is connected with a 50-Ω-coaxial cable from each side. The $S_{11}$-$S_{21}$ simulation and measurement comparison are plotted in Figure 1c. From the figure, it is noticed that the BALUN is well matched from 1.2 to 5 GHz with $S_{11}$ less than −10 dB. The measured $S_{21}$ curve has a difference of approximately −0.3 dB from the simulated curve, and this is caused by the additional connected BALUN. Generally, the BALUN frequency response is adequate for appropriate feeding of a spiral antenna.

### 2.2. Archimedean Spiral Antenna Design

**Figure 2:**
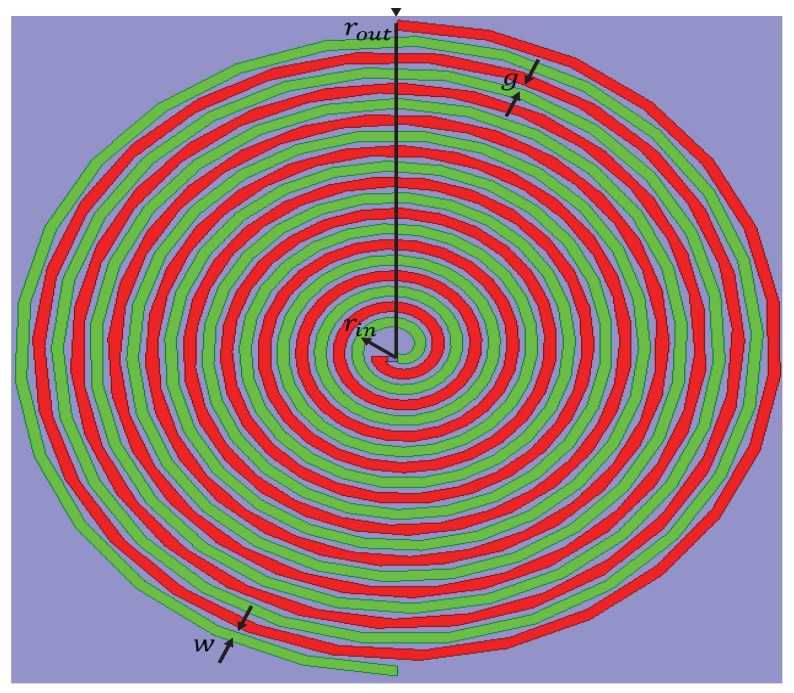
Layout of the antenna structure.

A two-arm Archimedean spiral antenna can be considered as a dipole, the arms of which are wrapped into the shape of an Archimdean spiral (Figure 2). An Archimedean antenna is a planar broadband-frequency-independent antenna structure and constructed easily using printed circuit-board techniques. The antenna is based on the classical geometry equation that describes the Archimedean structure [33] and is given by,

(1) $$\begin{array}{c} r=r_{o}\times \phi +r_{in}\\ r=r_{o}\times \left(\phi -\pi \right)+r_{in} \end{array}$$

Each arm of the ASDA is linearly proportional to angle ϕ and has a proportionality factor (defined the flare-out angle), the flare ratio $r_{o}$ is given by

(2) $$\begin{array}{c} r_{o}=\frac{g+w}{\pi }, \end{array}$$

where g is the inter-spacing between adjacent lines, and w is the strip width. The $r_{in}$ is the inner radius of the spiral antenna.

The antenna geometry is determined based on the lower- and higher-band frequency boundaries. The antenna radiates from an active region where the circumference of the spiral equals one wavelength. Each arm of the spiral is constituted, based on (1), 180° out of phase, so that the currents at complementary or opposite points on each arm of the spiral add in phase in the far field when the circumference of the spiral is one wavelength. The low- and high-frequency operating points are determined theoretically by the outer and inner radius, respectively (given in (3)). In practice, the low-frequency point is slightly shifted from the value predicted by (3) owing to the reflections from the end of the spiral. Moreover, the high-frequency limit is less than what can be found by using (3) owing to the feed region effects [34].

(3) $$\begin{array}{c} r_{out}=\frac{c_{o}}{2\times \pi \times f_{H}\times \sqrt{\varepsilon_{eff}}}\\ r_{in}=\frac{c_{o}}{2\times \pi \times f_{L}\times \sqrt{\varepsilon_{eff}}} \end{array}$$

In (3), $c_{o}$ represents the speed of the electromagnetic signal in free space and is equal 3×10${}^{8}$ m/s, and $\varepsilon_{eff}$ is the effective dielectric constant of the substrate. The dimensions of the antenna geometry are listed in Table 1, with total number of turns = 17.

### 2.3. Antenna Experimental Results

**Figure 3:**
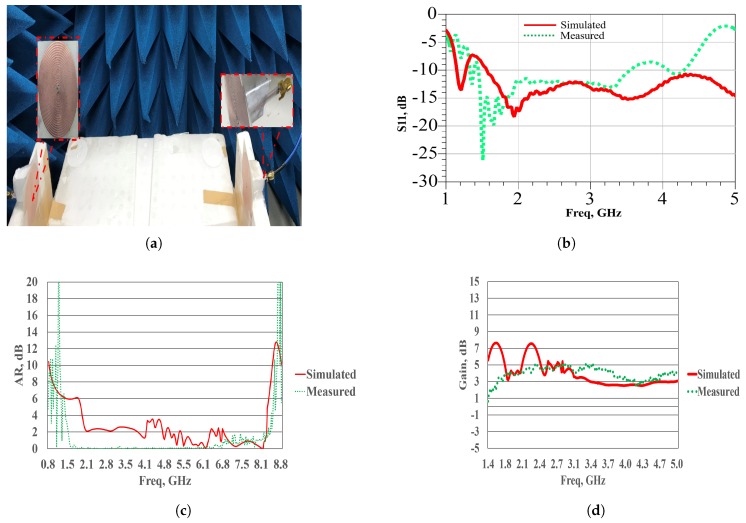

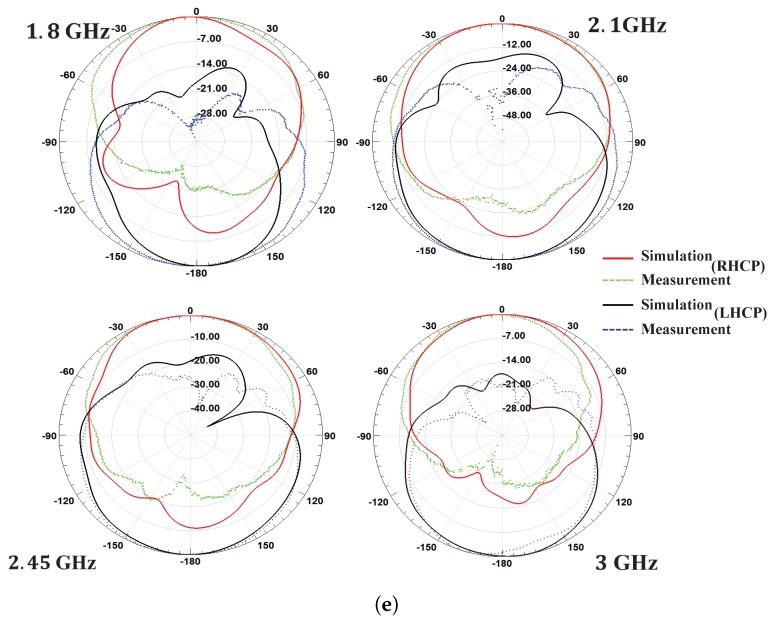
Antenna simulation and measurement performance characteristics, (**a**) Measurement set-up, (**b**) Input reflection coefficient $S_{11}$, (**c**) Axial ratio AR, (**d**) Realized gain, and (**e**) E-plane radiation pattern.

The ASDA is fabricated on a commercial low-cost substrate with relative dielectric constant $\varepsilon_{r}$ = 4.4, loss tangent 0.02, and thickness 0.8 mm. The proposed antenna has a compact size of 58 × 55 × 0.8 mm${}^{3}$. Both the simulation and measurement results are presented, demonstrating the UWB characteristics of the proposed antenna for wireless communication applications. A prototype of the proposed antenna is fabricated and tested. The antenna measurement set-up is given in Figure 3a. The measurement is performed inside the anechoic chamber.

The simulated and measured reflection coefficients are shown in Figure 3b. The overall −10 dB impedance band is from 1 to 5 GHz and above. It can be observed that the input impedance has real value 50 Ω and an imaginary part of approximately 0 Ω. Thus, it indicates a well impedance matching condition to the coaxial input port.

Figure 3c displays the simulated and measured axial ratio results of the antenna. It is noted that from 1.5 to 8.5 GHz, the antenna axial ratio is below 1 dB, and this implies that the cross-polarization ratio is below −30 dB. The gain versus frequency variation is illustrated in Figure 3d. It has an average value of 4.5 dBi with a maximum measured value of 5 dBi. The gain deteriorates at frequencies above 5 GHz owing to the mismatch with BALUN. Figure 3e shows the normalized radiation patterns of the antenna. Good right-handed circular polarization (RHCP) and left-handed circular polarization (LHCP) patterns with low cross polarization are obtained. The simulated and measured results are in good agreement. The radiation patterns are plotted at different in-band frequencies, namely, 1.8, 2.1, 2.45, and 3 GHz.

## 3. DR Design

**Figure 4:**
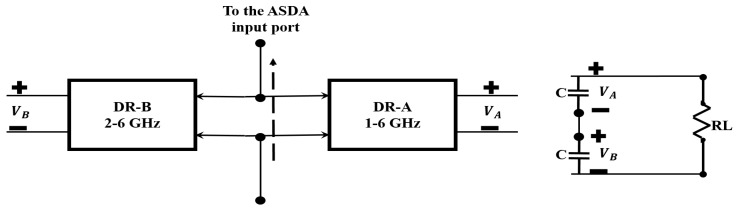
(**Left**) Block diagram of the two DRs adopted for the maximization of the rectifier operating frequency bandwidth. (**right**) Tiding of $V_{A}$ and $V_{B}$ for the connection of the terminal load.

Because the diode nonlinearity, input power levels, input signal frequency, and output load resistance are leading to performance degradation of the rectifier over a wide operating frequency band, a pair of DRs (DR-A and DR-B) is used for maximizing the operating bandwidth as shown in Figure 4. The DR design is inspired from a previous work [30,35]. In this communication, BALUN is removed, allowing a direct connection to the ASDA input. Consequently, the impedance matching bandwidth is maximized and rectifier performance is maintained across a broad bandwidth.

**Figure 5:**
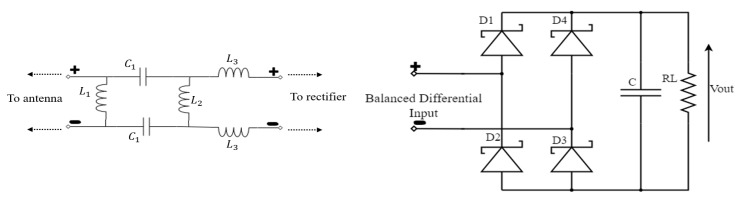
Voltage dual-DR architecture composed of four Schottky diodes and connected to the output RC circuit. On the left is the matching circuit, and on the right is the voltage doubler configuration.

Many rectifier configurations have been proposed, for instance, diode in series [36], diode in shunt [37], diodes in bridge [38], and diodes in voltage doubler[39]. The most common rectifier structure used for energy harvesting applications is the voltage doubler. Although this topology offers twice the input voltage, it does not provide a balanced output voltage. Therefore, this architecture is modified to realize a DR as shown in Figure 5. The closed-form mathematical equation of the DR was accurately deduced and analyzed in [40]. This circuit is composed of four diodes (D1, D2, D3, and D4), and each pair is conducting during one half cycle of the input signal. While diodes D1 and D3 are conducting during the positive half cycle of the input signal, diodes D2 and D4 are conducting during the negative half cycle. The pumped voltage is stored in output capacitor C, with the load connected across it. The Skyworks SMS7630 Schottky diode is used, and the package parasitics are considered during the simulation. A parasitic inductance value of Lp = 1.15 nH and parasitic capacitance value of Cp = 0.25 pF provided by the manufacturer are added to the design schematic.

**Figure 6:**
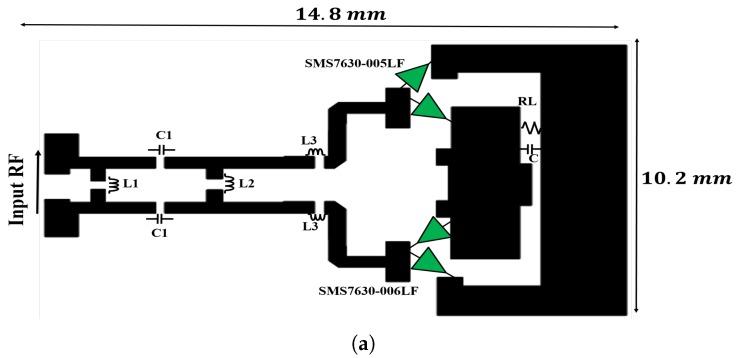

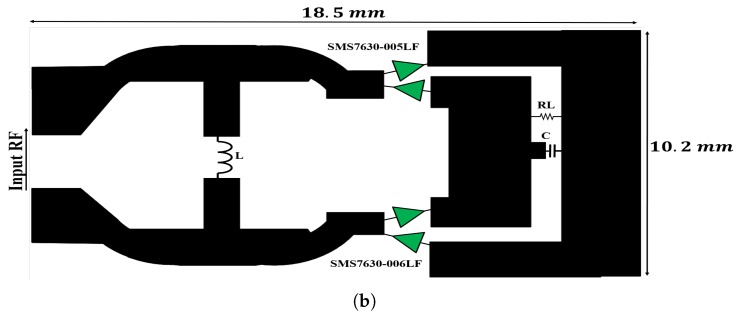
Topology of both the DR-A and DR-B rectifier circuits. (**a**) Layout of rectifier DR-A, (**b**) Layout of rectifier DR-B.

**Figure 7:**
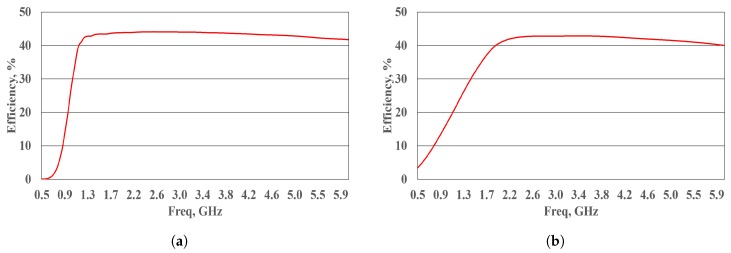
Harmonic balance simulation performance of the DR-A and DR-B rectifiers (**a**) DR-A response, (**b**) DR-B response. Both simulations were conducted at $P_{in}$ = 9 dBm and $R_{L}$ = 1 kΩ.

To achieve a broad bandwidth, two DRs are linked together. They are designated as DR-A and DR-B, as shown in Figure 6. DR-A is designed to operate across the band from 1–6 GHz. DR-A is manufactured using lumped elements L1, L2, L3, and C1, purchased from Murata Manufacturing Co. Ltd. (Nagaokakyo, Japan), and connected to two cascaded L-sections to provide broadband impedance matching. The value parameters (presented in Table 2) are obtained following a thorough optimization using the nonlinear harmonic balance simulation in the Agilent, ADS software. The input impedance of each rectifier is set as the ASDA input impedance $Z_{in}$. This impedance is obtained from the electromagnetic HFSS simulation of the ASDA without considering the BALUN connection. $Z_{in}$ is exported from the HFSS and introduced as the input port impedance of the rectifier circuit. The simulated rectifier performance of DR-A is exhibited in Figure 7a. As noted from the figure, DR-A has 40% conversion efficiency over the band from 0.95–6 GHz. These results are obtained at $P_{in}$ = 9 dBm and $R_{L}$ = 1 kΩ.

In comparison, DR-B is implemented using a single lumped element, L, connected in parallel to the DR circuit. This inductor offers good input matching characteristics from 2–6 GHz, as shown in Figure 7b, with more than 40% RF-DC conversion efficiency. The total output DC voltage obtained from both the schemes is given by,

(4) $$V_{dc}=V_{A}+V_{B}$$

where $V_{A}$ and $V_{B}$ are the balanced output voltages of DR-A and DR-B, respectively.

## 4. Experimental Measurement Results

The rectenna is built on a double layer FR4 substrate with thickness h=0.8 mm and dielectric constant $\varepsilon_{r}=4.4$ by using the print broad making equipment (MITS; FP-21T model 40).

**Figure 8:**
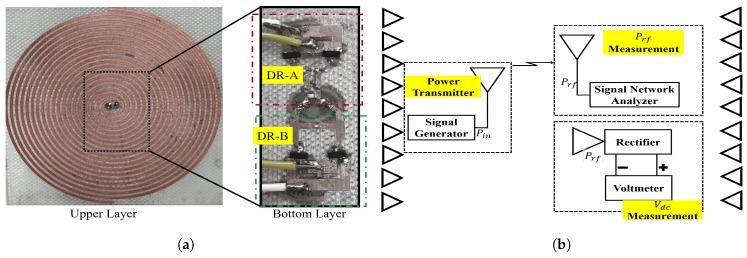
Photograph of the fabricated rectenna prototype and measurement setup, (**a**) Rectenna prototype showing both the upper layer (antenna) and bottom layer (rectifier); the rectifier is combined with two circuits DR-A and DR-B. (**b**) Measurement set-up with an antenna to determine the expected received power.

The rectenna prototype is displayed in Figure 8a. The conventional definition of the RF-DC conversion efficiency is the ratio of the amount of DC power at load $P_{dc}$ to RF input power collected by the antenna $P_{rf}$, and is given by,

(5) $$\eta =\frac{P_{dc}}{P_{rf}}=\frac{V_{dc}^{2}}{R_{L}\times P_{rf}}\times 100\%$$

**Figure 9:**
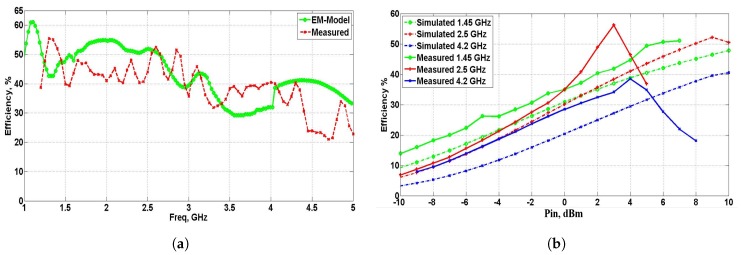
Rectenna measurement and simulated results. (**a**) Efficiency versus frequency at simulated $P_{in}$ = 9 dBm, $R_{L}$ = 1 kΩ. (**b**) Efficiency versus input power for $R_{L}$ =1 kΩ and at different input frequencies.

The measurements were performed to satisfy the far-field conditions. They were made with variation in the transmitted power and frequency, so that the received incident power changed according to the transmitted signal frequency, with the free-space losses being frequency-dependent. The absorbed received input power was estimated using an antenna connected to a signal network analyzer. The measurement set-up is presented in Figure 8b. The measurements were done inside an anechoic chamber represented by the triangles on the side walls. The power transmitter is emitting power into space with RF-power value of $P_{in}$. At the receiver side, we employed an antenna to measure the received power $P_{rf}$ and at the same time the rectenna is mounted on the same reception plane. The output characteristics of the rectenna is measured using a voltmeter. The rectenna output performance is calculated using (5), and the efficiency for different constraints is plotted in Figure 9. The response of the rectenna conversion efficiency with frequency is shown in Figure 9a. From the data shown, it is obvious that the results obtained from the measurements and EM model are in good agreement over the desired band with a maximum simulated efficiency at frequency 2 GHz. The measured efficiency is still more than 30% over the band of interest (1.2 to 5 GHz) and slightly reduced relative to the simulation results. The slight reduction in the measured efficiency for the higher frequencies inside the operating band is due to the resultant mismatching in the rectifier circuit at those frequencies. Indeed, the results deviation is coming from the inaccurate diode modeling over the suggested wideband of frequencies. To precisely model the diode over that broadband frequency, a circuit should be fabricated separately with only the diode and measure the real S-parameters along the desired band and then consider it during the simulation. Although such method is accurate, it would be time-consuming. In the manuscript, I supposed the model introduced in the datasheet. The efficiency versus input power is exhibited in Figure 9b. The curve is plotted at various input frequencies and fixed output load $R_{L}$ = 1 kΩ.

To fulfill the impact and contribution of the proposed rectenna design, a detailed comparison with state-of-the-art literature that discussed similar performance is presented in Table 3. The selected parameters for comparison are frequency bandwidth *f*, incident input power $P_{rf}$, achieved RF-DC conversion efficiency η, polarization type, circuit size, and the substrate used for the rectenna fabrication. In comparison with similar rectifiers, the rectenna presented in this paper achieves a very compact size that facilitate the integration with wireless sensor nodes. As an example, the current design provides a much reduced size than the circuit and rectenna presented in [20,22], respectively. Moreover, the proposed topology can offer a balanced output voltage suitable for supplying the integrated electronic circuit.

## 5. Conclusions

This paper presented a broadband RF energy harvester that exhibits efficient power conversion over the entire band from 1.2 to 5 GHz and has a simple two-layer design. The operating principles of the spiral antenna, rectifier, and matching network were presented in detail. The complete rectenna prototype was fabricated and tested by radiated measurements. More than 30% power conversion efficiency was measured across the desired frequency band, which represented the combined efficiency of the receiving antenna, impedance matching, and conversion by the rectifier into DC power at the load. Compared with a typical rectifier topology, the proposed DR exhibited improved RF-DC conversion efficiency, compact size, wider frequency range, and reduced sensitivity to the input received frequency. Further improvements in the rectenna could be realized by integrating the rectifier and antenna on the same metal plane. This could be obtained by the optimum design (strictly defining the minimum and maximum operating frequencies) of the antenna to save space for mounting the rectifier. 

## Figures and Tables

**Table 1:** Spiral antenna parameters.

Antenna Param.				Balun Param.			
**Param.**	**Value**	**Param.**	**Value**	**Param.**	**Value**	**Param.**	**Value**
$f_{U}$	10	$w_{1}$	2	$w_{1g}$	5	$l_{1}$	4
$f_{L}$	0.8	$w_{2}$	1.3	$w_{2g}$	3.5	$l_{2}$	8
$r_{in}$	3	$w_{3}$	1.1	$w_{3g}$	2	$l_{3}$	4
$r_{out}$	28	$w_{4}$	0.9	$w_{4g}$	1.5	$l_{4}$	10
*g*	0.65	$w_{5}$	0.8	$w_{5g}$	1	$l_{5}$	5
*w*	1	$w_{6}$	0.7	$w_{6g}$	0.8	$l_{6}$	5
$r_{o}$	0.64	$w_{7}$	0.5	$w_{7g}$	0.7	$l_{7}$	5
		$w_{8}$	0.4	$w_{8g}$	0.4	$l_{8}$	5

All units are given in mm except $f_{U}$ and $f_{L}$ in GHz.

**Table 2:** List of the DR-A and DR-B parameters.

Param.	L1	L2	L3	C1	L	C
Value.	33 nH	18 nH	2.7 nH	1 pF	17 nH	100 pF

**Table 3:** Comparison of this study with the relevant research reported in the literature.

REF	*f*, GHz	$P_{\mathrm{rf}}$	η, %	Polarization	Size, mm${}^{2}$	Substrate
[20]	2–18	1–100 μW/cm${}^{2}$	N/A	Dual CP	2800×2200	RO3850
[21]	0.25−3	10 dBm	33%	No antenna	150×10	Kapton
[22]	1–3.2	15 dBm	>50%	N/A	228.6×304.8	RO4003C
This work	1–5	9 dBm	>30%	Dual CP	58×55	FR4
